# Imprinted Proteins as a Receptor in Fluorescent Sensing Microplate Assay for Herbicide Determination

**DOI:** 10.3390/bios16030149

**Published:** 2026-03-03

**Authors:** Kirill Y. Presnyakov, Ivan S. Matlakhov, Ivan A. Reshetnik, Polina M. Ilicheva, Daria V. Tsyupka, Daria G. Koganova, Svetlana A. Mescheryakova, Tatyana Y. Rusanova, Mikhail V. Pozharov, Daniil D. Drozd, Pavel S. Pidenko, Irina Y. Goryacheva, Natalia A. Burmistrova

**Affiliations:** Institute of Chemistry, Saratov State University, Astrakhanskaya Street 83, Saratov 410012, Russiaivmatl.anohse@yandex.ru (I.S.M.); reshetnikia@sgu.ru (I.A.R.); tsyupkadv@mail.ru (D.V.T.); meshcheryakova.s.a@gmail.com (S.A.M.); pidenkops@sgu.ru (P.S.P.);

**Keywords:** herbicide, imazamox, imprinted proteins, alloyed quantum dots, turn-off assay, fluorescent microplate sensing

## Abstract

The manuscript describes an optical sensing microplate for the high-throughput screening of imidazolinone herbicides in soil extracts. As far as we know, imprinted proteins (IPs) specific to imidazolinone herbicides have not been synthesized and used as a recognition element for their solid-phase extraction before. Imprinted bovine serum albumin (BSA) and glucose oxidase (GOx) were synthesized in the presence of imazamox as a template and then these IPs were immobilized at the bottom of microplate wells. The sorption capacity (*Q*) of aminated silica nanoparticles modified by IPs (IP–BIS) was 6.38 mg g^−1^ while the imprinting factor (IF) equaled 2.6. The concentration of imazamox was determined by a “turn-off” solid-phase assay using alloyed CdZnSeS/ZnS quantum dots (QDs) as a component of fluorescent substrate. Alloyed CdZnSeS/ZnS QDs were stabilized in an aqueous phase by positively charged cysteamine that, as far we know, had not been used as this type of ligand before. Our method allows for determining the concentration of imazamox in the range of 0.5–9.2 μg mL^−1^, with a limit of quantification limit of quantitation (LOQ) equal to 0.45 μg mL^−1^ The sensing microplate enables parallel detection of up to 96 samples containing herbicides using standard fluorescence microplate readers or smartphones. The paper describes how such sensing microplates can be used for the analysis of artificially contaminated soil samples. The proposed approach combines pre-concentration of analyte at the IPs with its subsequent determination on a single analytical platform, thus allowing for both highly sensitive determination in laboratory conditions and mass screening in the field.

## 1. Introduction

Imidazolinones (IMIs) are a class of herbicides that are actively used worldwide by agro-industrial enterprises specializing in growing legumes, cereals, corn, rice, peanut crops and sunflower [1]. The extreme popularity of IMIs can be explained by a combination of their high herbicide activity and rather rapid photodegradation. Currently, one of the most common representatives of IMIs is imazamox [1,2]. It is moderately stable—its degradation time (${\mathrm{DT}}_{50}$) ranges from 20 to 500 days, depending on the characteristics of the soil, its microbiota and other environmental conditions [3,4,5]. Sunflower is one of the most important annual crops and is mostly grown for producing edible oil [6]. Russia is a world leader in sunflower production (16.9 million metric tons) [7], while the greatest quantity of sunflower sowing areas (225,673 Ha) is concentrated in the Saratov region [8].

The general problem of herbicide application (including imazamox) is the violation of recommendations for field treatment and introduction of excessive doses of herbicides into the soil [1,2]. Such improper application of imazamox results in accumulation of herbicide and its derivatives in soil [9,10] and affects non-resistant crops in the next farming cycle [11]. As IMIs are weak acids, they are most dangerous when introduced in excessive quantities into acidic soils. In the case of imazamox, its improper application may result in physiological and genetic changes in sunflower plants; accumulation of herbicide in sunflower sprouts; and, consequently, the reduced efficiency of photodegradation of the herbicide [3]. In normal conditions, the aforementioned negative factors are only temporary. However, extreme conditions (drought, changes in soil acidity, excessive soil moisture) significantly enhance the negative impact of imazamox [12]. This may result in a decreased sunflower development cycle, leading to reduced crop production and economic damage to regions where sunflower is the primary agricultural crop. Several countries have standards regulating the maximum permitted concentrations of imazamox (Table S1) in various agricultural products. In addition, prolonged exposure to imazamox is dangerous both to humans and the environment, thus necessitating regulation of its content in soils.

Soils are complex matrices; therefore, they require specific approaches to sample processing. Such approaches must be cost-effective and have a high extraction rate and selectivity to the target compound. In the case of IMI determination, the most common sample preparation methods are based on solid-phase extraction (SPE) [13]. Extraction of herbicides from soil is hampered by interference from humic compounds that may comprise up to 40% of all organic compounds present in natural waters [13]. Combination of SPE with artificial molecular recognition elements with high specificity to target compounds may serve as a potential solution to this problem. Molecularly imprinted polymers (MIPs) are a type of synthetic receptors that can be used both as a separate solid phase and as a modifier of the surface layer of various nano- and microsized sorbents added to improve the selectivity of SPE. Currently, there are MIPs (using 1-vinylimidazole, acrylamide or 4-vinylpyridine as a basis) that are specific to most IMIs (Table S2). The organic polymer-based MIPs have several key advantages, such as rather simple production techniques that utilize commonly available reagents, and the high physical and chemical stability of the resulting MIPs [14]. However, optimization of MIP synthesis conditions is very time-consuming, while the complete structure of the polymer matrix and the number of binding sites is hard to predict. Imprinted proteins (IPs) have several key advantages compared with synthetic organic polymers. Their synthesis does not require any toxic reagents, while the optimal protein matrix composition, the protein:template ratio and structure of potential dummy templates can be estimated through specialized software simulations [15,16]. Thus, IPs serve as a promising alternative to traditional MIPs [17].

High-performance liquid chromatography (HPLC) and gas chromatography with various kinds of detectors serve as primary tools for monitoring the concentration of imazamox in soil and food products [1,18]. However, development of highly effective determination techniques that require minimal sample processing and simple laboratory equipment remains an actual problem. Such techniques have to combine the extraction and concentration of target molecules with the detection of analytical signals at a single analytical platform. The most promising platforms that can be used for such task are polystyrene microtiter plates (microplates). The material of microplates can be easily modified with receptor elements, while the fact that polystyrene is transparent for wavelengths greater than 350 nm allows for using UV–Vis and fluorescence spectroscopy to register analytical signals [19,20].

Fluorescence-based analytical methods are characterized by a low limit of quantitation (LOQ), simplicity of detection, and applicability to many different analytical methods and labels (fluorescent molecules and nanoparticles), thus making these methods good candidates for potentially highly effective analytical techniques. High photostability is one of the key requirements for fluorescent labels, with quantum dots (QDs) having the best photostability among all kinds of fluorescent labels [21]. Imazamox has good water solubility; therefore, its concentration can be determined in aqueous soil extracts. The best QDs that can be used for analysis in aqueous media are alloyed CdZnSeS/ZnS QDs because their hydrophilization does not reduce their quantum yield or stability of the colloid solution [22].

This paper describes an imazamox determination technique based on a “turn-off” assay using a fluorescence-sensing microplate. Bovine serum albumin (BSA)- and glucose oxidase (GOx)-based IPs were used as molecular recognition elements. Imazamox was determined using a “turn-off” assay with IPs pre-concentration (IPs–QDsTOA) by novel hydrophilic alloyed CdZnSeS/ZnS QDs modified with cysteamine (CYS), a positively charged ligand.

## 2. Materials and Methods

### 2.1. Materials

BSA, imazamox (98%), glutaraldehyde (50% wt. aqueous solution), (3-aminopropyl)-triethoxysilane (99%), cadmium (II) acetate (99.995%), zinc acetate (99.99%), zinc stearate (technical grade), elemental selenium (powder), elemental sulfur (powder), trioctylphosphine (90%), 1-octadecene (90%), oleic acid (90%) and cysteamine hydrochloride (97%) were purchased from Merck (Merck KGaA, Steinheim, Germany); GOx was purchased from Trading house Biopreparat LLC, Obolensk, Russia. Phosphate-buffered saline (0.1 M PBS, Rosmedbio LLC, Saint Petersburg, Russia) was prepared from tablets. Hydrochloric acid, sodium hydroxide, dimethyl sulfoxide (DMSO, 99%) and acetonitrile were purchased from Reakhim (Reakhim LLC, Moscow, Russia). Nunc-Immuno MicroWell 96-well microtiter plates (flat-bottom, high binding capacity) were purchased from Merck KGaA, Steinheim, Germany.

All solutions were made using double-distilled water treated via a Milli–Q system (Millipore, Burlington, MA, USA). The purity of all other chemicals was analytical grade. Aerosil 200 (Evonik GmbH, Essen, Germany) was used as a carrier for preparation of aminated silica nanoparticles modified by IPs (bioinorganic sorbent modified with IPs, IP–BIS). A 5000 MWCO Dispo-Biodialyzer (Biodialyzer, JET Bio-Filtration Co., Guangzhou, China) was used for the centrifuge concentration to carry out the purification of IPs.

### 2.2. Imprinting and Purification of Proteins

Protein imprinting was performed in accordance with the procedure described in [23,24,25]. A total of 1 mg of protein was dissolved in 1 mL of Milli–Q-grade water and incubated for 10 min. Then the protein solution was titrated with 0.1 M HCl until pH 2 and kept in these conditions for 20 min. The optimal concentration of template was found using software modeling, as described in [15]. To produce the protein–imazamox associate, 100 μL of imazamox solution was added to the protein solution and incubated for 20 min while the solution was stirred continuously. Capture of conformation of the protein–imazamox associate was performed by adding 100 μL of 0.1% *v*/*v* glutaraldehyde solution and subsequent filtration of the reaction medium to pH≈8. The produced IPs were purified by centrifugal concentration (5000 MWCO Dispo-Biodialyzer). IP samples were placed into a filter, then an eluent (i.e., PBS) was added until the total volume of mixture equaled 10 mL. After centrifugation of the solution (3600 rcf, 4 °C, 5 min), the eluent that passed through the filter was gathered for further testing. The procedure was repeated until full purification of the IPs (final volume of eluent was 15 mL per 1 mL of IPs). The produced IP solutions were concentrated until their absorbance corresponded to the absorbance of stock solutions (0.1 mg mL^−1^ of native proteins. The purified IPs were stored in dark glass vials in a freezer at 4 °C. Non-imprinted proteins (nIPs) were produced without the template and used as a control.

### 2.3. Synthesis of QDs

Synthesis of alloyed CdZnSeS/ZnS QDs was performed in accordance with a modified previously published procedure [22]. In brief, 6.2 mg and 112 mg of cadmium and zinc acetates, respectively, were placed into a three-necked flask; then 2.3 mL of oleic acid and 5 mL of 1-octadecene were added to it. The reaction medium was evaporated for 40 min at 150 °C and a residual pressure of $1\times 10^{-2}$ mbar (or less) to remove water and acetic acid. The reaction medium was then filled with inert gas (argon) and heated to 310 °C. After reaching the required temperature, 0.15 mL of 1.5 M solutions of sulfur and selenium in trioctylphosphine were injected into the reaction medium; then 300 μL of shell precursor solution (a mixture of 2 mL of 1-octadecene, 3000 mg of zinc stearate and 0.25 mL of 1.5 M sulfur solution in trioctylphosphine) was added dropwise to the medium five times at 5 min intervals. After 5 min had passed since the addition of the last portion of precursor solution, the reaction was stopped by putting the three-necked flask into room temperature water. The obtained colloid particles were deposited by the centrifugation and addition of ethanol until the formation of opalescence; sedimented particles were then dissolved in 2.5 mL of chloroform. To modify the surface of QDs with positively charged ligands, a solution containing 15 mg of CYS in 20 μL of DMSO was added to 50 μL of the solution of produced colloid particles in chloroform. The reaction medium was thoroughly mixed and kept in ultrasonic bath (at 40 kHz) for 20 min. During this process, the phases separated and QDs (colored part) completely transitioned to DMSO. Modified QDs were deposited by the addition of 50 μL of ethanol and centrifugation (7000 rcf) for 5 min. The resulting sediment was rinsed with chloroform several times and re-deposited by centrifugation in order to remove the excess of unbounded ligand, any byproducts and DMSO. After that, the sediment was left to air dry and then dissolved in 1 mL of double-distilled water.

### 2.4. Computational Details

The process of formation of protein–imazamox associates was studied via computer modeling. The procedure and methodological aspects of the computer simulation were previously described by Ilicheva et al. [15]. X-ray structures of BSA (PDB ID: 4F5S, https://www.rcsb.org/structure/4F5S (accessed on 3 February 2025), resolution: 2.47 Å) and GOx (PDB ID: 1CF3, https://www.rcsb.org/structure/1CF3 (accessed on 3 February 2025), resolution 1.90 Å) were taken from Protein Data Base [26]. Protein structures were modified before the simulations by adding hydrogen atoms and missing side chains to amino acids, deleting solvent molecules and correcting the protonation state in order to simulate a more acidic medium (pH 3). Association of BSA with template molecules was studied by molecular docking algorithms implemented in AutoDock 4.2 [27].

### 2.5. Screening of Binding Potential of IPs

Evaluation of sorption characteristics of produced IPs was performed for IP–BIS synthesized as described by Fu et al. [28]. A total of 1 mL of working solutions of imazamox (1.5–200 μg mL^−1^) were added to 5 mg of IP–BIS and incubated while continuously stirred using rotation shaker for 1 h at room temperature. After incubation, the mixture was centrifuged (5000 rcf, 5 min), the supernatant was taken and tested for imazamox concentration using HPLC–UV (LC–20AD, Shimadzu Co., Kyoto, Japan). HPLC–UV was performed with Waters Spherisorb ODS2 C18 column (150 mm × 4.6 mm, 5 μm; Waters Corporation, Milford, MA, USA) in isocratic mode at a flow rate of 0.6 mL min^−1^ and a constant temperature of 45 °C with mobile phase A (H_3_PO_4_, 0.1% *v*/*v*., pH 2.8) and mobile phase B (acetonitrile) taken at a ratio of 55:45 (A:B). The sorption capacity (*Q*) of IP–BIS was evaluated using Equation (1):

(1) $$Q=\frac{(c_{in}-c_{fin})\times V}{W},$$

where $c_{in}$ and $c_{fin}$ are the initial and final concentrations of imazamox, respectively; *V* is the sample volume; and *W* is the weight of sorbent.

The imprinting factor (IF) was evaluated using Equation (2):

(2) $$IF=\frac{\tan\alpha_{IP–BIS}}{\tan\alpha_{nIP–BIS}},$$

where $\tan\alpha_{IP–BIS}$ and $\tan\alpha_{nIP–BIS}$ are the slope ratios of Henry regions of adsorption isotherms for imazamox extraction by IP–BIS and BIS modified with non-imprinted protein (nIP–BIS), respectively.

### 2.6. “Turn-Off” Assay with IPs Pre-Concentration

**Figure 1:**
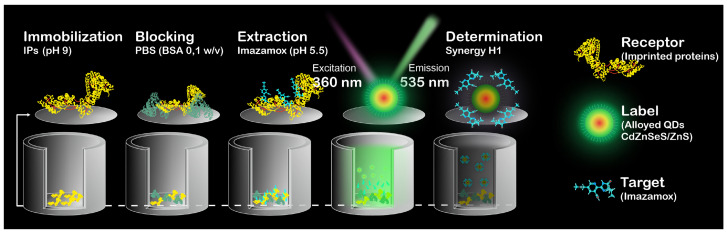
Schematic representation of IPs–QDsTOA.

The concentration determination of imazamox using IPs–QDsTOA was performed in accordance with the schematic provided in Figure 1. IPs were immobilized within microplate wells (pH 9, 100 μL/well, 37 °C, 4 h), blocked with a buffer solution (0.1 M PBS) that contained BSA (0.1% *w*/*v* BSA, 200 μL/well) and kept at 37 °C for 1 h. Working solutions of imazamox (0.12–160 μg mL^−1^, 0.1 M PBS, 100 μL/well) were added to microplate wells and incubated for 1 h at 37 °C. After each step, the microplate wells were rinsed thrice with a 0.1 M PBS solution (300 μL/well) to remove any unbound IPs, blocking buffer solution and non-specifically bound imazamox. Desorption of imazamox from binding sites was performed with Milli–Q (pH 5.5, 50 μL/well, 37 °C, 30 min); then QDs were added (initial solution diluted by 40 times, $A_{360}=0.1$ a. u., Milli–Q, pH 5.5, 50 μL/well) and kept in the wells at 37 °C for 30 min. The fluorescence spectra of the solutions were registered with Synergy H1 Hybrid Multi-Mode Reader (BioTek Inst., Winooski, VT, USA) using the following parameters: $\lambda_{\mathrm{ex}}=360$ nm, emission range: 480–600 nm, step: 2 nm and measurement height = 6.5 mm. The calibration curve was plotted for the dependence of QDs fluorescence intensity (for $\lambda_{\max}=535$ nm) on the herbicide concentration.

The LOQ was calculated using Equation (3):

(3) $$\begin{array}{c} \begin{array}{c} LOQ=\frac{10\sigma }{\tan\alpha } \end{array} \end{array}$$

where σ is the standard deviation of the control fluorescent signal, and tanα is the slope of the linear range of the sigmoidal standard curves.

### 2.7. Sample Preparation

Soil samples were collected at fields (without sunflower) within the municipality of Saratov. Soil samples were ground into powder, and a portion of the sample (10 g) was extracted with Milli–Q (pH 5.5, 30 mL) at a rotation shaker in the course of 30 min. The supernatant was taken after centrifugation (4000 rcf, 25 °C, 15 min). The extract was titrated with HCl until pH 3 and centrifuged to remove humic acids. Before IPs–QDsTOA, the extract was diluted five-fold with water (pH 5.5). Absence of imazamox in the soil extract was validated by HPLC–UV.

## 3. Results and Discussion

The proposed imazamox preconcentration technique was implemented using BSA- and GOx-based IPs as molecular recognition elements. Previously, it was found that IPs can be effectively used as substitutes for antibodies in zearalenone [29] and kwakhurin [24] determination via solid-phase assays with IPs preconcentration (IPs–CSPA) on microplates. We believe that development of microplate-based determination techniques is the most promising approach to soil analysis. All existing IPs–CSPA techniques require production of conjugates of target compounds with enzymes (horseradish peroxidase) or fluorescent (fluorescein isothiocyanate, 6-aminofluorescein) labels. The process of conjugation includes time- and labor-intensive stages of extraction and purification of target compounds, thus complicating these techniques. Therefore, it is important to develop analysis techniques that do not require conjugates to generate analytical signals.

This can potentially be achieved by developing procedures for determination of target compounds at microplates using “turn-off” assays based on QDs fluorescence quenching. Fluorescence quenching is already used in various high-sensitive analytical techniques applicable to a wide range of compounds—from metal ions to biological molecules [30,31,32,33,34,35]. Selectivity and sensitivity are achieved by selecting the appropriate composition of QDs core and targeted functionalization of its surface with specific ligands.

Imazamox is a nicotinic acid derivative where the imidazole fragment is attached to a carbon atom next to a nitrogen in the pyridine ring (position 2). The imidazole fragment has two substitutes in position 4 of the ring (isopropyl and methyl groups) and a carboxylic group in position 5, while nicotinic acid also has a methoxymethyl group attached to position 5 of the pyridine ring (Figure S1). According to analysis of its structure and previously published data [35], the imazamox concentration can potentially be determined using a “turn-off” assay with QDs modified with a positively charged sulfur-containing ligand, i.e., CYS.

### 3.1. Imprinting of Proteins

Protein imprinting consists of four main stages: protein protonation and change of conformation, formation of a protein–template associate, capture of associate’s conformation in alkaline medium and purification of formed binding sites. Previously, it was shown that IPs produced at pH 2 had the best sorption characteristics [23]. The second stage of the imprinting process was studied via molecular modeling. Blind docking was carried out for the protein matrix in the presence of multiple imazamox molecules. Molecular modeling showed that there were nine and twelve potential binding sites for imazamox molecules on BSA and GOx, respectively (Figure S2). Interactions between imazamox and the protein matrix during imprinting predominantly involved binding through hydrophobic sites, as well as through hydrogen bonds (including water-mediated ones). Analysis of the ligand–protein associate structure showed that the potential binding sites were saturated with hydrophobic and charged amino acids (Table 1). The optimal calculated protein:imazamox ratios were 1:45 and 1:60 for BSA and GOx, respectively. These ratios were subsequently used to produce IPs.

**Figure 2:**
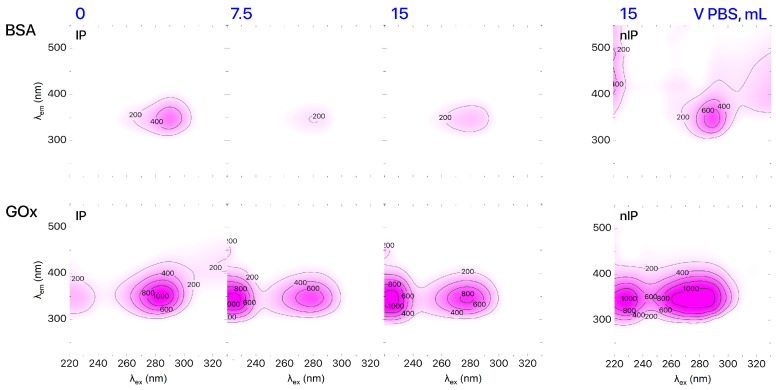
EEMs of IPs based on BSA and GOx during purification.

The final stage of imprinting is removal of template molecules from formed binding sites. Receptor purification was confirmed by plotting the excitation–emission matrix (EEM) using the results of 3D fluorescent spectroscopic analysis of the IPs solution. 3D fluorescence spectra were recorded via a Cary Eclipse (Agilent Technologies Inc., Santa Clara, CA, USA) fluorescent spectrometer. According to the EEM data (Figure 2), the pattern of IPs solution (1 mL) after elution with 15 mL of PBS was identical to the pattern produced for nIPs. UV–Vis spectroscopy (UV–1800 spectrophotometer, Shimadzu Co., Kyoto, Japan) was used to determine the concentration of the template in the eluent and evaluate the efficiency of template extraction from IP binding sites. We found that the IP purification rates were 91% and 98% for IPs based on BSA and GOx, respectively. It should be mentioned that there are some problems with the investigation of morphology of IPs. Despite that, we can see that imprinted polymer retains the native protein morphology at pH 3 as shown by TEM image provided in [23].

### 3.2. Synthesis and Characterization of Positive Charged Alloyed QDs

As far as we know, modification of alloyed QDs CdZnSeS/ZnS with CYS to provide a positive charge to the surface of nanocrystals has never been done before. The principle of modification is based on a previously designed procedure of modification of alloyed QDs with functional thiols [22]. Due to the ionic nature of CYS, the procedure includes an additional stage where the modifying agent is dissolved in DMSO, as the ligand exchange reaction occurs in the liquid phase.

**Figure 3:**
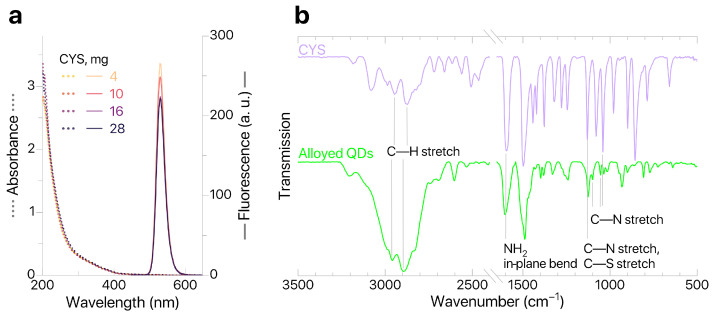
(**a**) QDs absorption and fluorescence; (**b**) ATR–FTIR spectra of CYS and QDs.

Modification of alloyed QDs was performed in an excess of CYS. As the weight of modifying agent per standard portion of QDs increased from 4 to 28 mg, no significant changes in the absorption spectra of standard sample aliquots were observed (Figure 3), thus confirming that the nanoparticles were mostly transferred to the aqueous phase. The relative photoluminescence quantum yield (PLQY) was calculated (Cary Eclipse, Agilent Technologies Inc., Santa Clara, CA, USA) against the fluorescent dye coumarin C153. The ζ-potential of produced QDs@CYS was evaluated (Zetasizer Ultra Red Label, Malvern Instruments Ltd., Malvern, UK) using electrophoretic light scattering and characterized at IPs–QDsTOA conditions (Milli–Q pH 5.5); the results are shown in Table 2.

According to Table 2, the increased weight of CYS results in an increased ζ-potential and decreased PLQY. The sample with the highest ζ-potential values (16 mg of CYS) was used for further studies. However, it should be noted that there is no significant variation in properties of QDs for all studied weights of CYS. This means that application of an excessive amount of modifying agent (more than 4 mg per standard portion of QDs) has minimal effect on the colloidal and optical characteristics of modified QDs@CYS. To further confirm that ligands were successfully replaced by CYS, we studied the QDs@CYS samples with ATR–FTIR spectroscopy (FT–801 infrared Fourier spectrometers with attenuated total reflection attachments, Simex, Novosibirsk, Russia). The ATR–FTIR spectrum of QD sample contains characteristic bands corresponding to stretching vibrations of C—H bonds (2959 and 2895 ${\mathrm{cm}}^{-1}$), NH_2_ in-plane bending (1603 ${\mathrm{cm}}^{-1}$), stretching vibrations of C—N bonds (1126, 1100, 1055, 1036 ${\mathrm{cm}}^{-1}$) and stretching vibrations of C—S bonds (1100 ${\mathrm{cm}}^{-1}$). This confirms that ligands were successfully replaced by CYS. A sample containing 16 mg of CYS (with the greatest ζ-potential value) was tested for quenching of QDs fluorescence in the presence of imazamox and used for further development of IPs–QDsTOA.

### 3.3. IPs Sorption Characteristics and Optimal Conditions for Imazamox Extraction

**Figure 4:**
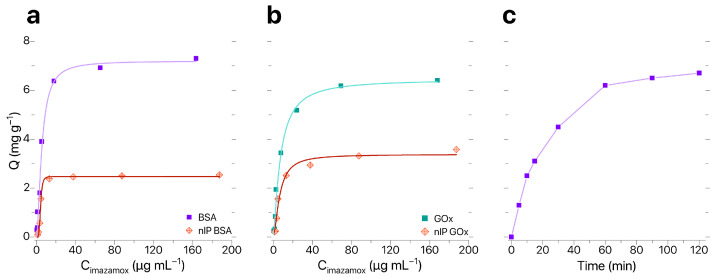
(**a**,**b**) Sorption isotherms and (**c**) dynamic sorption curve for imazamox by IP–BIS.

Sorption characteristics of IP–BIS modified by synthesized IPs were evaluated in accordance with the procedure described in [28]; the results of evaluation are shown in Figure 4a,b. Analysis of sorption isotherms obtained for IP–BIS modified with BSA- and GOx-based IPs showed that sorption capacity *Q* changed linearly until the concentration of imazamox equaled 25 μg mL^−1^. Maximal equilibrium values of *Q* and IF were observed for samples of IP–BIS modified with BSA-based IPs (*Q* = 6.38 mg g^−1^, IF = 2.6). The optimal time of imazamox extraction was 60 min (Figure 4c).

### 3.4. “Turn-Off” Assay with IPs Pre-Concentration

**Figure 5:**
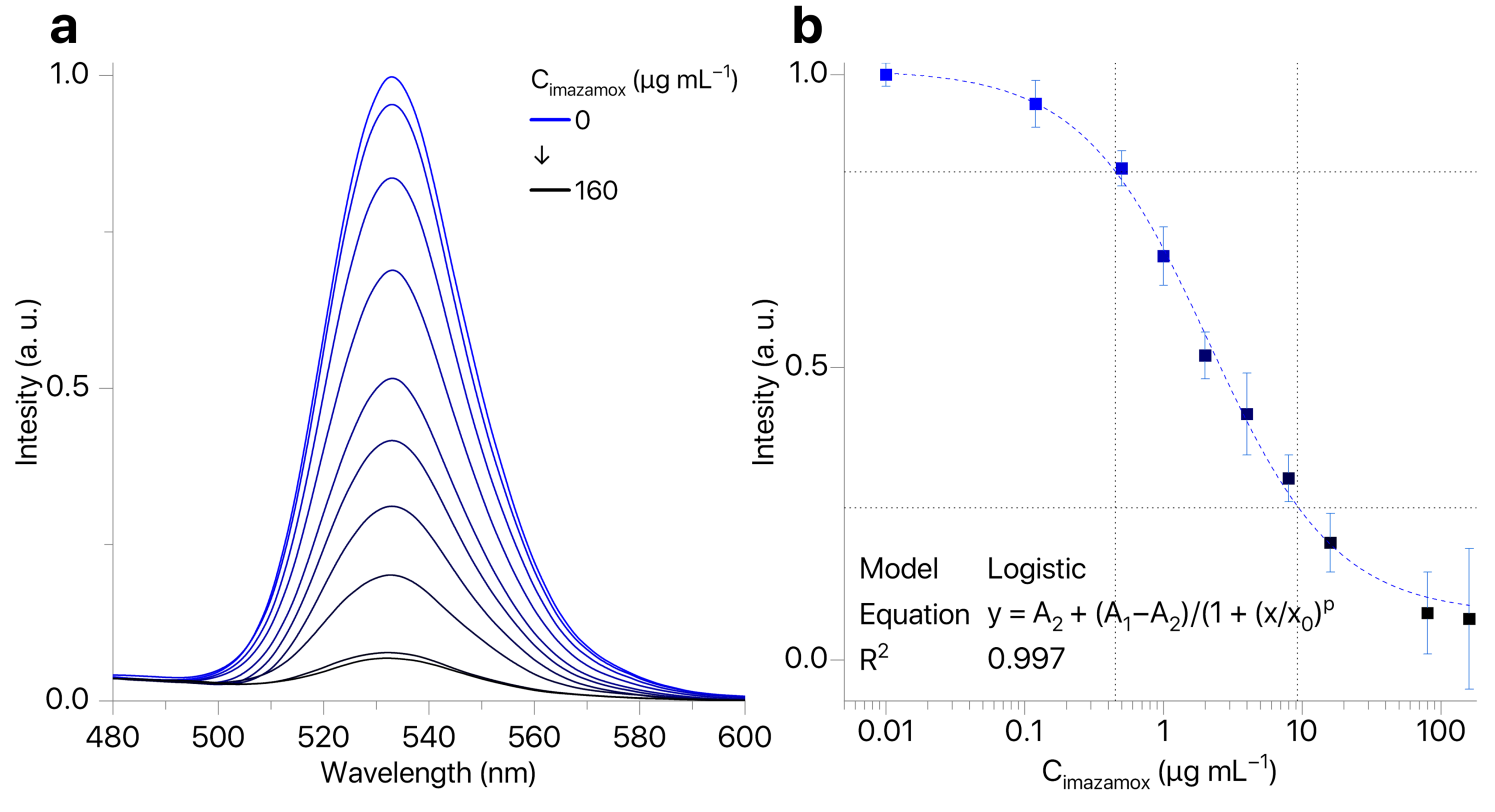
(**a**) QD fluorescence spectra in the presence of imazamox (0.12–160 μg mL^−1^); (**b**) calibration curve for imazamox detection by fluorescence quenching of QDs (n=3).

The presence of imazamox results in quenching of QDs fluorescence (Figure 5a). At the same time, the shape and position of the QDs emission peak remained the same while the intensity of QDs fluorescence depended on concentration of imazamox (Figure 5b). Our technique allows for determining the concentration of imazamox in the linear range of 0.5–9.2 μg mL^−1^ (LOQ = 0.45 μg mL^−1^). Thus, we have shown the possibility of development of a technique for determining the concentration of imazamox based on quenching of QD fluorescence.

To understand the mechanism of the fluorescent response, we conducted an additional study of imazamox’s impact on the intensity of fluorescence of alloyed CdZnSeS/ZnS QDs stabilized by dihydrolipoic acid (ζ-potential = −35±5 mV). Quenching of fluorescence after addition of QDs was the same as for the clean solvent (Milli–Q water), even after 30 min of their addition. We think that in the case of QDs stabilized by CYS, their surface becomes available for electrostatic attraction to imazamox molecules due to a carboxylic group present in its structure. Electrostatic attraction between QDs and imazamox brings them close enough to each other for energy transfer between QDs surface and electron delocalization centers of herbicide (pyridine and imidazole rings). This mechanism is corroborated by other studies on luminescence quenching of negatively charged QDs in similar systems [34,36]. The exact nature of this energy transfer requires additional studies.

In the case of soil samples, the matrix effect on the QD fluorescence is a very important factor. To evaluate the matrix effect, we studied the QD fluorescence in the presence of “AminoFulvin” (ElementRa LLC, Saratov, Russia), a commercial ameliorant containing ≈90 mg mL^−1^ of humic compounds in total. According to our observations, a concentration of humic compounds at a level of 0.001 mg mL^−1^ scarcely affects the intensity of QDs fluorescence (quenching ≈3%), while addition of 1 mg mL^−1^ of humic compounds resulted in a significant decrease in QD fluorescence (quenching ≈93%). We studied the impact of free amino acids present in soil—aspartic acid (Asp), glutamic acid (Glu) and glycine (Gly)—on QD fluorescence. Incubation of 1 mg mL^−1^ solutions of QDs resulted in a reductions in QD fluorescence by ≈40, ≈25 and ≈15%, respectively. Therefore, the application of QDs for imazamox determination is significantly affected by the soil matrix and soil samples require preparation before conducting an analysis.

The developed technique utilizes protein molecules as receptors. It is known [37] that proteins can associate on the surface of QDs, leading to decreased availability of their binding sites and, as a result, inability of interaction between the fluorophore and quenching agent. Thus, we evaluated the effect of IPs on QD fluorescence and found that the introduction of stock solutions of IPs (≈0.1 mg mL^−1^) based on BSA and GOx resulted in decreases in QD fluorescence by 35% and 25%, respectively. The concentration of IPs in solutions diluted from stock solutions by a factor of 100 is comparable with the concentration of IPs at the surface of the microplate after immobilization; such concentrations do not significantly decrease the QD fluorescence (±5–7%).

The efficiency of imazamox extraction by IPs depends on several factors (Table S3). The durations of periods of IPs immobilization, blocking, analyte extraction and imazamox incubation were chosen in accordance with data provided in [16,32]. We also assessed the effect of the IP concentration on the extraction efficiency. Solutions of IPs based on BSA were diluted by a factor of 10, 20, 40, 80 or 120, immobilized in microplate wells, and used to extract imazamox from its 160 μg mL^−1^ solution. The maximal recovery rate (≈96%) was observed for IPs based on BSA diluted by a factor of 80. However, the observed recovery rate had low reproducibility, which can be attributed to the uneven immobilization of IPs at the surface of microplate wells. Higher concentrations of IPs (solutions diluted by factors of 10 and 20) resulted in decreases in the recovery rate by up to 14% and 26% (for the solutions of IPs based on GOx and BSA diluted by a factor of 10, respectively), which can potentially be attributed to association of IPs molecules at the surface of the microplate, resulting in the formation of an additional layer of protein that decreases the availability of some binding sites. It seems that a greater area of GOx molecules leads to the formation of more stable associates and protein layers, thus significantly reducing the recovery rate of imazamox (14%) compared with BSA (26%) when using solutions with the same concentration (diluted by a factor of 10). Reproducible results were achieved for solutions diluted by a factor of 40; for BSA-based IPs, the recovery rate equaled 89±3%, while for GOx-based IPs, it equaled 70±5%. Thus, solutions of BSA-based IPs diluted by a factor of 40 were used for further optimization of the analysis conditions. We studied the effect of pH on the efficiency of IPs immobilization at the surface of the microplate. Recovery of imazamox increases as the pH increases from acidic (pH 3, HCl, ≈71%) to neutral (pH 7.4, PBS, ≈82%) and, finally, low-alkaline conditions (pH 9, NaOH, ≈89%). This can probably be explained by the fact that the final IP conformation capture occurs in mildly alkaline conditions (pH 8) during the synthesis. We also studied the effect of concentration and composition of blocking buffer solutions (PBS containing 1, 0.5, 0.1 and 0% wt. BSA) on the recovery of imazamox by IPs. Maximal recovery rates (87±3% and 91±2%) were observed for PBS solutions containing 0.5 and 0.1% wt. of BSA, respectively. A further increase in BSA concentration decreased recovery rate to ≈80%. IPs without blocking solution were capable of extracting greater quantities of imazamox (recovery rate 99±15%), but the reproducibility of experimental results was very low and led to false negative results of analysis and risk of extraction of matrix components from actual samples. For further studies, we chose the following conditions for imazamox extraction: BSA-based IPs solution diluted by a factor of 40 from stock solution (pH 9, 4 h) and PBS containing 0.1% wt. of BSA as a blocking solution (1 h); the durations of process stages were as follows: imazamox extraction—1 h, imazamox desorption—30 min and QDs incubation with analyte—30 min.

Estimated optimal conditions were used to assess the effect of matrix humic compounds on IPs–QDsTOA. Incubation of humic compounds (1 mg mL^−1^) by IPs did not lead to quenching of the QDs’ fluorescence (signal intensity equaled 96% from control). Simultaneous incubation of humic compounds (1 mg mL^−1^) and imazamox (20 μg mL^−1^) led to quenching of QDs comparable with quenching by model solutions of imazamox. Also, incubation (Figure S3) of free amino acids (Asp, Glu, Gly) did not lead to quenching of QDs’ fluorescence (maximal quenching by ≈3.5% was observed for Asp). We also studied the potential for imazamox extraction (10 μg mL^−1^) by microplates surface modified with nIP and IPs based on BSA. The intensity of QDs fluorescence decreased by ≈4% for nIP and by 72% for IPs. Thus, non-specific sorption has only a minor impact on detection results. Considering the known structural similarity IPs, we believe that this receptor will have an affinity with other IMIs. The experimental data was used to plot a calibration curve for the determination of imazamox in model soil extracts (concentration of humic compounds = 1 mg mL^−1^, imazamox concentraion range: 0.12–160 μg mL^−1^). Our technique allows for determining the concentration of imazamox within the linear range of 0.5–9.8 μg mL^−1^ (LOQ = 0.44 μg mL^−1^. Analytical characteristics obtained by IPs–QDsTOA are similar to those obtained from the addition of model solutions of imazamox to QDs.

### 3.5. Analysis of Soil Extract Samples

The developed IPs–QDsTOA was tested using a set of six (6) artificially contaminated soil extract samples (three were prepared from 10 cm below ground level and three—from 20 cm below ground level), which were analyzed in triplicate. Soil extracts were contaminated by imazamox at six levels: 0.25, 0.50 and 2.50 μg mL^−1^ for samples from 10 cm below ground level and 0.70, 1.40 and 7.00 μg mL^−1^ for samples from 20 cm below ground level to imitate the changes of imazamox concentration in soil. Comparison of IPs–QDsTOA and HPLC–UV results is provided in Table 3. It shows good agreement between methods. The samples were additionally spiked and the outcome validated the results for IPs—QDsTOA using spike/recovery analysis (Table 3). The recovery for IPs–QDsTOA was 93–101% and the relative standard deviation ($S_{r}$) did not exceed 5.2%.

## 4. Conclusions

The paper describes an approach to imazamox determination using IPs–QDsTOA. We developed a technique for producing alloyed CdZnSeS/ZnS QDs stabilized in aqueous phase by positively charged ligands (CYS). As far as we know, synthesized BSA- and GOx-based IPs specific to imazamox have never been synthesized before. The application of IPs for extraction of imazamox from soils helped to significantly (by 32 times) decrease the interference from humic compounds regarding quenching of QDs fluorescence. The developed technique allows for determining imazamox in the linear range of 0.5–9.8 μg mL^−1^ (LOQ = 0.44 μg mL^−1^). The technique demonstrates good agreement (average 94%) between reference method (HPLC–UV) and IPs–QDsTOA for determination of the concentration of imazamox in artificially contaminated soil extracts.

The technique proposed in this paper serves as a proof of principle that can be used to apply IPs as novel receptor elements for “turn-off” analysis. Potentially, this technique can be adapted for the concentration determination of a wide range of low-molecular water-soluble compounds present in various environmental objects and biological fluids. However, it should be noted that the efficiency of application of IPs–QDsTOA mostly depends on the efficiency of quenching of the QDs’ fluorescence in the presence of a chosen analyte.

## Figures and Tables

**Table 1:** Ligand interactions between protein matrix and imazamox molecules at pH 3.0.

Ligand	BSA	GOx
1	Arg198, Ser201, Hydroph. (Ala200, Trp213)	Glu356, Asn473
2	Glu399, Arg427, Hydroph. (Tyr400)	Arg263, Arg537, Arg545
3	Hydroph. (Pro117, Leu122, Ile141)	Arg113, Trp133, Hydroph. (Cys164, Cys206)
4	Tyr333, Hydroph. (Ala306, Phe308, Phe373)	Hydroph. (Leu85, Ala86, Tyr509, Phe511)
5	Ser469, Lys471, Asp493, Hydroph. (Val414)	Polar (Ser121, Thr124, Asn398), Charged neg. (Glu397, Asp120)
6	Thr507, Phe508	Charged neg. (Asp416)
7	Hydroph. (Tyr340, Val380)	Asn43, Lys282
8	Hydroph. (Leu103, Cys199, Cys245, Il202)	Lys570, AspH573, Hydroph. (Ala574)
9	HisH366, Hydroph. (Val314, Cys315, Tyr369)	Gln153, Tyr182
10	−	Asp319, Hydroph. (Met305)
11	−	Arg335, Arg512
12	−	HisH78, Tyr249

**Table 2:** Properties of alloyed CdZnSeS/ZnS@CYS obtained with different amount of CYS.

CYS, mg	PLQY, %	ζ-Potential, mV
4	42	27±2
10	42	25±2
16	38	39±9
28	33	31±8

**Table 3:** Comparison of imazamox determination in soil samples via HPLC–UV and IPs–QDsTOA and validation by spike/recovery analysis (n=3, P=0.95).

Spiked,	Detected, µg mL^−1^		Spiked,	Detected, µg mL^−1^	Recovery,	$S_{r}$,
µg mL^−1^	*HPLC–UV*	*IPs–QDsTOA*	µg mL^−1^	*IPs–QDsTOA*	%	%
*10 cm below ground level*						
0.25	0.24	nd	0.69	0.68	99	3.5
0.50	0.49	0.48	0.94	0.92	98	1.7
2.50	2.44	2.51	2.94	2.94	100	5.2
*20 cm below ground level*						
0.70	0.69	0.67	1.14	1.15	101	4.1
1.40	1.36	1.29	1.84	1.72	93	3.6
7.00	6.85	6.63	7.44	7.05	95	2.7

nd—negative result.

## Data Availability

All data generated or analyzed during this study are included in the published article and its Supplementary Materials.

## Supplementary Materials

The following supporting information can be downloaded at: https://www.mdpi.com/article/10.3390/bios16030149/s1, Table S1: Maximum permissible levels of imazamox in cereal plants in different countries; Table S2: Molecularly imprinted polymers selective to herbicides; Table S3: Influence of assay conditions to imazamox recovery rate (%) at IPs modified microplate surface; Figure S1: Imazamox structure; Figure S2: Complex protein–imazamox at pH 3.0; Figure S3: Influence of free amino acids in soil for fluorescence of QDs with and without IPs modification of microplate. References [13,38,39,40,41,42,43,44,45,46] are cited in the Supplementary Materials.

## Abbreviations

The following abbreviations are used in this manuscript:

Asp	Aspartic acid
BSA	Bovine serum albumin
CYS	Cysteamine
DMSO	Dimethyl sulfoxide
EEM	Excitation–emission matrix
Glu	Glutamic acid
Gly	Glycine
GOx	Glucose oxidase
HPLC	High-performance liquid chromatography
IF	Imprinting factor
IMI	Imidazolinones
IPs	Imprinted proteins
IP–BIS	Bioinorganic sorbent modified with IPs
IPs–QDsTOA	“Turn-off” assay with IPs pre-concentration
LOQ	Limit of quantitation
MIPs	Molecularly imprinted polymers
nIP	Non-imprinted proteins
ODE	1-Octadecene
PBS	Phosphate saline buffer
PLQY	Relative photoluminescence quantum yield
QDs	Quantum dots
SPE	Solid-phase extraction
