# Supplementary material for: Imprinted Proteins as a Receptor in Fluorescent Sensing Microplate Assay for Herbicide Determination
Source: Biosensors (Basel). 2026 Mar 3;16(3):149. doi: 10.3390/bios16030149 (PMC13024476; doi:10.3390/bios16030149)
Supplement: Supplementary file 1 [file biosensors-16-00149-s001.zip › biosensors-3972574-supplementary.pdf]

Imprinted Proteins as a Receptor in Fluorescent Sensing Microplate Assay for Herbicides Determination

Supplementary material

Kirill Yu. Presnyakov[1], Ivan S. Matlakhov[1], Ivan A. Reshetnik[1], Polina M. Ilicheva[1], Daria V. Tsyupka[1], Daria G. Koganova[1], Svetlana A. Mescheryakova[1], Tatyana Yu. Rusanova[1], Mikhail V. Pozharov[1], Daniil D. Drozd[1], Pavel S. Pidenko[1], Irina Yu. Goryacheva[1], Natalia A. Burmistrova[1]*

[1] *Institute of Chemistry, Saratov State University, Astrakhanskaya Street 83, Saratov, 410012, Russia.*

* — corresponding author, e-mail address: naburmistrova@mail.ru

List of Tables

S1 Maximum permissible levels of imazamox in cereal plants in different countries . . . i
S2 Molecularly imprinted polymers selective to herbicides . . . ii
S3 Influence of assay conditions to imazamox recovery rate (%) at IPs modified microplate surface . . . iii

List of Figures

S1 Imazamox structure . . . iv
S2 Complex protein–imazamox at pH 3.0 . . . v
S3 Influence of free amino acids in soil for fluorescence of QDs with and without IPs modification of microplate . . . vi

Table **S1**: Maximum permissible levels of imazamox in cereal plants in different countries

Country	Culture	Concentration limit	Ref.
Russia	Sunflower seeds	0.004 $mg\,m^{-3}$	[38]
USA	Wheat	0.3 ppm	[39]
China	Wheat	0.05 $mg\,kg^{-1}$	[40]
Australia	Barley	0.02–0.04 $mg\,kg^{-1}$	[41]
EU	Sunflower seeds	0.30 $mg\,kg^{-1}$	[42]

Table **S2**: Molecularly imprinted polymers selective to herbicides

Monomer / Cross-linked agent	Detectable substances	Substrate	LOD	Linear range	Recovery	Ref.
1–VN	Imazapyr	n/a	0.09 $\mu g\,L^{-1}$	0.29–200.0 $\mu g\,L^{-1}$	86–107 %	[13]
	Imazapic	=	0.06 $\mu g\,L^{-1}$	0.21–200.0 $\mu g\,L^{-1}$	=	
	Imazethapyr	=	0.04 $\mu g\,L^{-1}$	0.15–200.0 $\mu g\,L^{-1}$	=	
	=	fCBPE	0.03 $\mu mol\,L^{-1}$	0.10–70.00 $\mu mol\,L^{-1}$	96.3–105.7 %	[43]
AA / TRIM	=	CPR	15 $ng\,g^{-1}$	0.1–5.0 $\mu g\,mL^{-1}$	91.1–97.5 %	[44]
	=	Fe_3O_4@SiO_2	2.13 $\mu g\,L^{-1}$	5–100 $\mu g\,L^{-1}$	87.7–102 %	[45]
4–VP / EGDMA	=	SPME fiber	0.16 $\mu g\,mL^{-1}$	0.50–50 $\mu g\,mL^{-1}$	87.5–123 %	[46]
	Imazameth	=	0.16 $\mu g\,mL^{-1}$	=	70.0–99.9 %	
	Imazamox	=	0.070 $\mu g\,mL^{-1}$	=	60.0–87.9 %	
	Imazapyr acid	=	0.16 $\mu g\,mL^{-1}$	=	63.1–87.0 %	
	Imazaquin acid	=	0.29 $\mu g\,mL^{-1}$	1.0–100 $\mu g\,mL^{-1}$	81.2–99.5 %	

All papers in the table used **imazethapyr** as a template.
List of acronyms: =—similar value, **1–VN**—1-vinylimidazole, **4–VP**—4-vinylpyridine, **AA**—acrylamide, **CPR**—chloromethylation polystyrene resin, **EGDMA**—ethylene glycol dimethacrylate, **fCBPE**—functionalized carbon black paste electrode, **LOD**—limit of detection, **MA**—methacrylic acid, **SPME**—solid phase microextraction, **TRIM**—trimethylolpropane trimethacrylate

Table **S3**: Influence of assay conditions to imazamox recovery rate (%) at IPs modified microplate surface

		**Recovery rate, %**	
		BSA based IPs	GOx based IPs
IPs dilution (pH 8, blocking buffer 0.5 % wt. BSA)	10	31±7	14±8
	20	64±6	38±4
	40	89±3	70±5
	80	96±12	86±10
	120	93±14	92±15
IPs immobilization pH (IPs 40, blocking buffer 0.5 % wt. BSA)*	3.0	71±4	nd
	7.4	82±2	nd
	9.0	89±1	nd
BSA content in blocking buffer, % wt (IPs 40, pH 9)**	0	99±15	nd
	0.1	91±2	nd
	0.5	87±3	nd
	1.0	80±2	nd

nd—Negative result, *,**—The optimal condition for immobilization pH and blocking buffer were obtained for BSA based IPs as a result in recovery rate at IPs dilution investigation

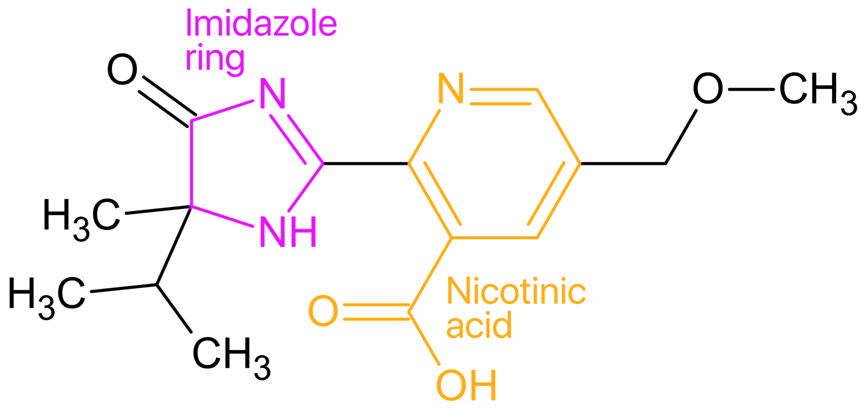

Figure **S1**: Imazamox structure

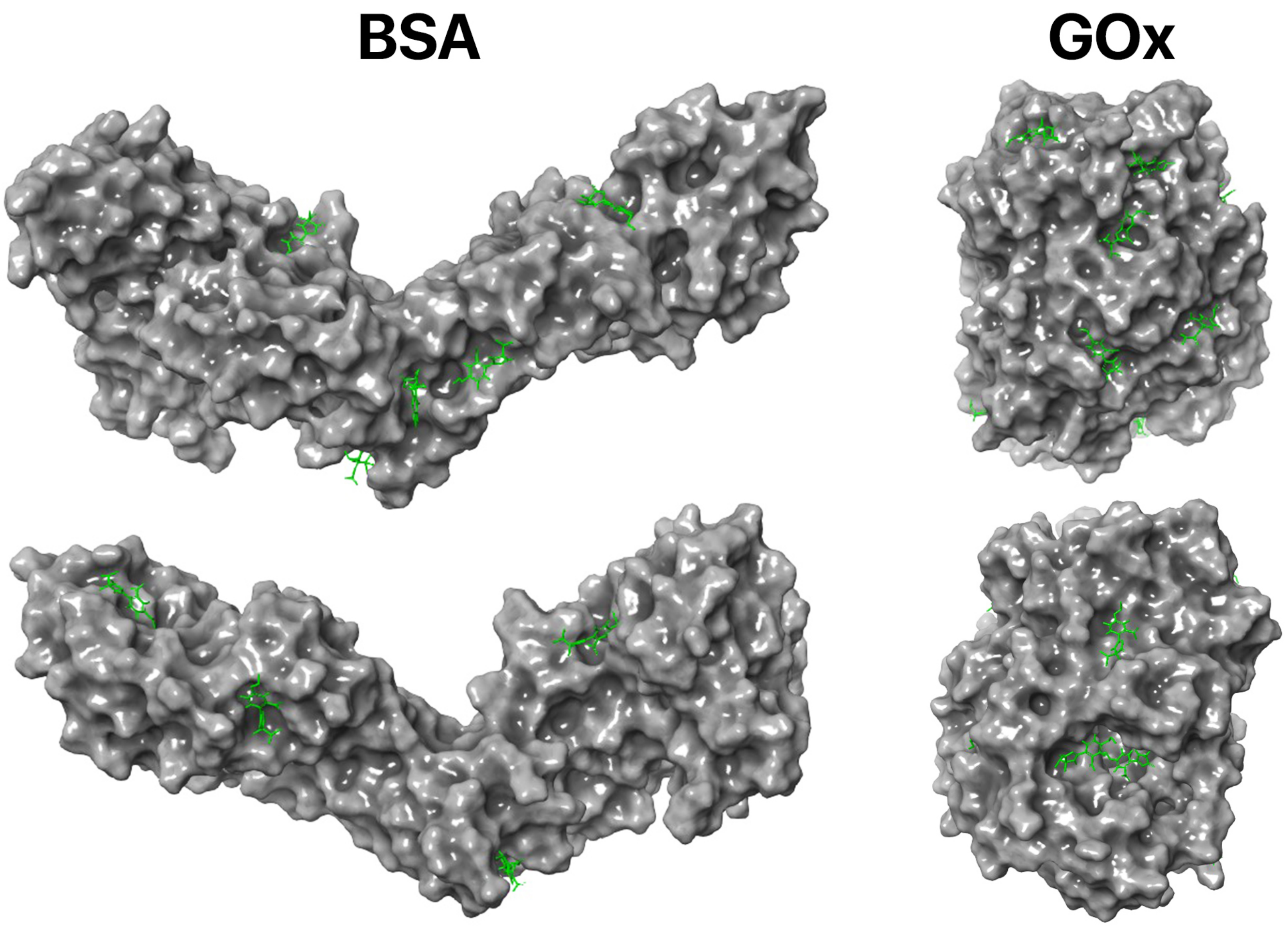

Figure **S2**: Complex protein–imazamox at pH 3.0

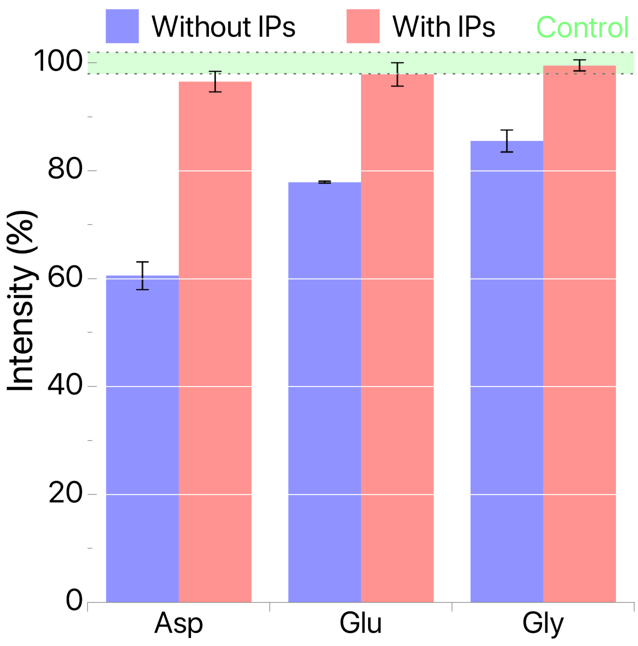

Figure **S3**: Influence of free amino acids in soil for fluorescence of QDs with and without IPs modification of microplate
